# Optical and Dielectric Properties of BaF_2_:(Er,Yb) Co-Doped Crystal

**DOI:** 10.3390/ma18091915

**Published:** 2025-04-23

**Authors:** Marius Stef, Carla Schornig, Gabriel Buse

**Affiliations:** 1 Faculty of Physics, West University of Timisoara, Bd. V. Parvan 4, 300223 Timisoara, Romania; marius.stef@e-uvt.ro (M.S.); carla.schornig76@e-uvt.ro (C.S.); 2 Institute for Advanced Environmental Research, West University of Timisoara, 300086 Timisoara, Romania

**Keywords:** fluoride crystals, erbium, optical spectroscopy, luminescence

## Abstract

A BaF_2_ single crystal co-doped with Er^3^⁺ and Yb^3^⁺ was grown by the vertical Bridgman technique and investigated for its optical and dielectric properties. Judd–Ofelt analysis yielded intensity parameters Ω_2_ = 0.59, Ω_4_ = 0.38, and Ω_6_ = 0.27 (×10^−20^ cm^2^), with a quality factor χ = 1.41, indicating strong radiative transitions. Under UV and near-UV excitation, emissions at 321, 405, 518, and 536 nm were observed, with radiative lifetimes ranging from 1.1 to 3.4 ms. A single dielectric relaxation process was identified, with activation energy of 0.58 eV and associated with trigonal NNN dipoles. The NNN dipole concentration was estimated at ~2.5 × 10^18^ cm^−3^. These results support the suitability of Er^3^⁺,Yb^3^⁺ co-doped BaF_2_ crystals for luminescent and dielectric applications in advanced photonic materials.

## 1. Introduction

Luminescent materials utilizing rare-earth (RE) ions continue to captivate the scientific community due to their intriguing properties and hold significant relevance for a wide array of emerging applications. Rare-earth-doped fluorite materials are well known as active media for solid-state lasers from ultraviolet up to middle infrared spectral region [1,2,3,4]. Due to the up-conversion phenomenon of fluorites doped or co-doped with rare earth ions, they are being studied for their applications as optical temperature sensors [5,6,7,8,9,10], solar radiation converters [11], or scintillators [12,13]. Although many types of materials co-doped with Er and Yb ions have been studied [14,15,16,17,18,19,20,21,22], there are few studies concerning the optical and dielectric properties investigation of Er-doped and Yb-codoped BaF_2_ crystal. Recently, N. Li et al. [23] reported a color-tuned up-conversion luminescence of BaF_2_:Yb^3+^, Er^3+^ 1D nanostructures containing nanofibers, nanobelts, and hollow nanofibers under excitation on 980 nm. S. Balabhadra et al. have reported an up-conversion fluorescence upon selective excitation of Yb^3+^ ions at 980 nm, resulting in a strong visible emission from ^2^H_11/2_ (blue), ^4^S_3/2_ (green) and ^4^F_9/2_ (red) multiplets [24]. However, there is limited information regarding the luminescent properties of double-doped BaF_2_:(Er,Yb) crystal [25], but for up-conversion luminescence only. BaF_2_ crystal have a cubic structure in the Fm-3m space group, with Ba^2+^ ions suitable for rare-earth doping. For a low dopant concentration, the charge compensation takes place through interstitial F^−^ ions in the next-nearest neighbor (NNN) lattice positions, resulting in trigonal site symmetries [3]. Higher dopant concentrations lead to the formation of complex defects and clusters in the crystal. The presence of local charge compensation results in the formation of electric dipoles, which exhibit relaxations detected as dielectric absorption. Dielectric relaxation measurements of Er^3+^ ions in BaF_2_ crystals were recently reported [26]. A single dielectric relaxation has been observed, characterized by activation energy of 0.54 eV which is attributed to the trigonal (C_3v_) centers. Similarly, the dielectric spectra of Yb^3+^ doped BaF_2_ crystals have shown a relaxation peak around 0.54 eV also assigned to the NNN dipoles (namely Yb^3+^–F(*i*) compensating defect with C_3v_ site symmetry) [27]. It is important to mention that in both cases, the dopant concentration is less than or equal to 0.5 mol%. With the increase in dopant concentration, the number of isolated centers decreases, giving rise to new aggregates or clusters. Using the method described by Nicoara et al. [26], the number of NNN dipoles can be calculated. It is known that certain trivalent rare earth ions (such as Eu, Sm, Ho, Tm, and Yb) can dissolve in the BaF_2_ crystal in both trivalent and divalent state. The presence of Yb^2+^ ions is revealed in the UV absorption spectrum and the presence of Yb^3+^ ions is in the near-IR. The Yb^2+^ ions replace Ba^2+^ ions, require no charge compensation, exhibit cubic site symmetry, and do not contribute to the dielectric spectra [27]. Compared to CaF_2_ or SrF_2_, BaF_2_ allows efficient charge compensation through interstitial fluorine ions located in next-nearest-neighbor positions (NNN), leading to well-characterized trigonal (C_3v_) dipoles. This makes it a model system for studying dielectric relaxation associated with NNN dipoles [27]. Moreover, BaF_2_’s low phonon cutoff energy and high transparency in the UV–MIR range enhance its suitability for investigating the luminescence phenomena in RE^3^⁺/RE^2^⁺ co-doped systems [11,19]. Despite its advantages, detailed studies on the dielectric and photoluminescent behavior of Er^3^⁺,Yb^3^⁺ co-doped BaF_2_ crystals remain limited, particularly in relation to defect–dipole dynamics, which we aim to address in this work. Both dielectric and absorption spectra can provide information about the nature and site symmetry of the defects. Spectroscopic properties of Yb^2+^ and Yb^3+^ ions and, separately, Er^3+^ ions doped in BaF_2_, crystals have been investigated, particularly for applications as up-conversion luminescent materials in the UV–VIS–NIR spectral range [3,12,13,28,29,30]. There are few papers related to the luminescence properties of Er/Yb double-doped crystals under excitation in the visible range (downshifting emission), but no study reported for BaF_2_:(Er,Yb) crystals. In 2019, Liu et al. [31] reported that PbF_2_:(Er,Yb) phosphors show a green emission, around 550 nm, and red emission at 660 nm, under excitation at 376 nm, which correspond to ^4^S_3/2_→^4^I_15/2_ and ^4^F_9/2_→^4^I_15/2_ transitions of Er^3+^ ions, respectively. Red, green, and blue up-conversion emissions were also observed in Er/Yb double-doped Ba_2_LaF_7_ nanocrystals [32]. Kaczmarek et al. [33] reported a near infrared emission, at room temperature, around 1540 and 978 nm of (Er^3+^,Yb^3+^):LaF_3_ nanoparticles under excitation at 378 nm (direct excitation of ^4^G_11/2_ level). On the other hand, by direct excitation at 380 nm, a broad and intensive emission, with maximum around 410 nm, was observed in Bi_2_ZnOB_2_O_6_:Yb^3+^/Er^3+^ single crystal which correspond to to the ^3^P_1_ → ^1^S_0_ transition of Bi^3+^ ions from the host crystal [34].

The focus of this paper is to investigate the spectroscopic and dielectric properties of double-doped BaF_2_:(Er,Yb) crystal. To achieve this objective, optical absorption and photoluminescence (PL) measurements were obtained, and the Judd–Ofelt (JO) model was used to obtain information about the luminescence properties of the crystal. The obtained theoretical values were compared with those reported by other authors. The dielectric measurements were utilized to investigate the nature of charge compensating defects arranged in a dipolar configuration. Within this analysis, the dielectric absorption unveiled the relaxation process of these dipoles. The study identified a singular type of charge compensating defect, specifically, the isolated C_3v_ site symmetry. The examination of these physical properties of the low concentration double-doped BaF_2_:(Er,Yb) crystal holds significance not just from a scientific perspective but also for practical applications.

## 2. Materials and Methods

**Figure 1:**
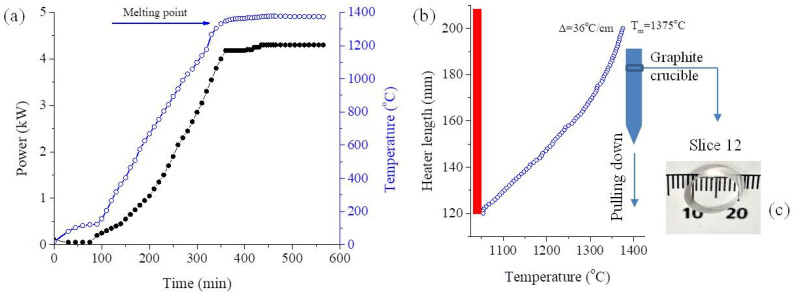
(**a**) Preparation stage of the Bridgman setup to reach the melting temperature in the graphite heater. (**b**) The temperature gradient, measured at the bottom of the crucible during the crystallization process. (**c**) The cleaved sample (slice 12).

Double-doped BaF_2_:(0.05 mol% YbF_3_, 0.2 mol% ErF_3_) crystal was grown in our Crystal Growth Laboratory by vertical Bridgman method using a shaped graphite furnace [35]. The doping concentrations mentioned pertain to the inclusion of ErF_3_ and YbF_3_ into the molten substance. The crystal was grown under a vacuum of approximately 10^−1^ Pa in a crucible made of spectrally pure graphite. We utilized crushed BaF_2_ optical UV–VIS windows sourced from Crystran Ltd., Dorset, UK (derived from 99.99% BaF_2_ powder) as our initial material. To this raw material, the specified quantity of ErF_3_ and YbF_3_ (purchased from Merck, Dramstadt, Germany, 99.99% purity) was added. Figure 1a shows the preparation stage of the Bridgman setup for reaching the rated electrical power of 4.3 kW on the graphite heater. This electrical power is required to achieve the melting temperature of the raw material (1375 °C). A period of three hours at the 4.3 kW is required in order to stabilize the melt. Figure 1b illustrates the typical temperature distribution measured at the bottom of the crucible using an S-type thermocouple. The crucible, with the melt, goes down in the heater with a pulling rate of 4 mm/h. In the first hour of the growth process, the temperature gradient was 36 °C/h. The grown crystal has approximately 6 cm long and 10 mm in diameter. The crystal was cleaved along the (111) crystallographic direction into several slices. For this study, a 2.33 mm thick slice was selected from the top of the crystal (slice 12). It exhibits transparency and is devoid of any visible inclusions or cracks, as depicted in Figure 1c.

The room temperature optical absorption spectra in the UV–VIS–NIR spectral range were recorded using a Shimadzu 1650PC, Schimadzu Corporation, Kyoto, Japan and Nexus 470 FTIR spectrophotometer, Thermo Fisher Scientific, Waltham, MA, USA The spectrophotometers use an automatic correction for baseline correction. The correction takes into account the effect of instrument noise and the light scattering due to the possible undesired particles in the sample. In order to measure the room temperature luminescence spectra, at room temperature, in the UV–VIS domain, the PerkinElmer LS55 spectrofluorometer, Perkin Elmer Inc., Waltham, MA, USA, was used. The room temperature luminescence spectra were obtained by excitation at two wavelengths, 290 and 378 nm, respectively, which correspond to the ^4^I_15/2_→^4^G_7/2_ and ^4^I_15/2_→^4^G_11/2_ transition of Er^3+^ ions [13]. The local charge compensations by pairing the trivalent rare-earth (RE^3+^) cations with interstitial F^−^(i) anions create electric dipoles, (RE^3+^-F^−^). These dipoles can reorient under a variable electric field. The reorientation process can be studied by various methods as anelastic relaxation, thermally stimulated depolarization, or dielectric relaxation measurements. The temperature and frequency dependence of the complex dielectric constant, ε* = ε_1_ − iε_2_ constitutes the dielectric spectra. The dielectric spectra, spanning ten audio frequencies, were obtained using the RLC Meter ZM2355 NF Corporation, Yokohama, Japan, across temperatures ranging from 150 K to 320 K. The real part of the dielectric constant, ε_1_, was derived from the measured capacitance C, while the imaginary part, ε_2_, was subsequently calculated through relation D = ε_2_/ε_1_, where D = tan θ is the dielectric loss. The measurements were conducted employing linear heating rates of 1 K/min. A sample cleaved from single crystal, polished to ~0.6 mm thick disk, was used, with Ag (Leitsilber) contacts facilitating the dielectric property assessments.

## 3. Experimental Results and Discussion

### 3.1. Optical Absorption Spectra

**Figure 2:**
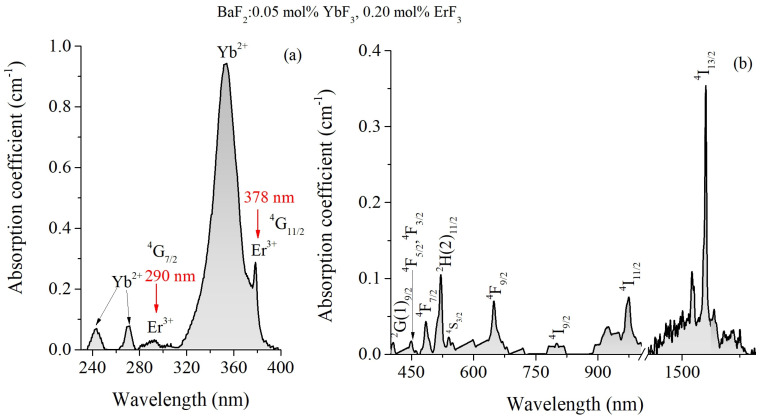
Optical absorption spectra of double-doped BaF_2_:(Er,Yb) crystal at room temperature in (**a**) 230–400 nm spectral region and (**b**) 440–1600 nm.

Figure 2a,b shows the absorption spectra of the selected sample. The absorption spectra show both the characteristic absorption bands of trivalent Er ions and those of Yb^3+^/Yb^2+^ ions, the latter resulting from the Yb^3+^→Yb^2+^ electric charge conversion, similar to that observed in CaF_2_:(Er,Yb) crystals [19]. The absorption peaks, denoting the transitions from the ground state ^4^I_15/2_ to the excited states of Er^3+^ ions, are specifically highlighted in Figure 2a,b. Also, in Figure 2a, the absorption bands of Er^3+^ ions around 290 and 378 nm, used for excitation during emission monitoring, are highlighted. Due to the charge compensation process, involving both rare earth ions, the energy levels of the RE ions split causing the formation of broad and structured absorption bands.

### 3.2. Judd–Ofelt Analysis

The application of the standard Judd–Ofelt analysis (J-O analysis) [36,37] facilitated the calculation of spectroscopic parameters based on optical absorption spectra. By utilizing a set of four absorption bands corresponding to specific Er^3+^ transitions from the ground level ^4^I_15_ to excited levels (^4^I_13/2_, ^4^F_9/2_, ^2^H(2)_11/2_, and ^4^F_7/2_), the J–O intensity parameters, namely Ω_2_, Ω_4_, and Ω_6_, were computed. The 980 nm absorption band was excluded due to overlapping transitions of Er^3+^ ions and Yb^3+^ ions (specifically, ^4^I_15/2_→^4^I_11/2_ for Er^3+^ ions and ^2^F_7/2_→^2^F_5/2_ for Yb^3+^ ions). Additionally, the absorption band around 378 nm (^4^I_15/2_→^4^G_11/2_ transition), characteristic of Er^3+^ ions, was excluded from the J–O analysis due to overlapping with the Yb^2+^ ion absorption band related to the 4f^14^(^1^S_0_)→4f^13^5d (^2^F_3/2_) transition.

In order to obtain the J-O parameters Ω_t_ (t = 2, 4, 6) and the measured line strength (see Table 1), we solved a set of four equations, using the Levenberg–Marquardt algorithm, corresponding to the four transitions. The experimental line strength has been determined using the following expression:

(1) $$S_{meas}=\frac{3\hbar c\left(2J+1\right)n(\lambda )}{8\pi^{3}N_{0}\lambda_{mean}e^{2}}\left[\frac{9}{{\left({n(\lambda )}^{2}+2\right)}^{2}}\int \alpha \left(\lambda \right)d\lambda \right]$$

where *J* is the total angular momentum quantum number of the initial state, *n*(λ) is the refractive index, *N*_0_ is the Er^3+^ ions concentration (added to the melt), λ_mean_ is the mean wavelength of the specific absorption bands, $\Sigma =\int \alpha \left(\lambda \right)d\lambda$ is the integrated absorption coefficient as a function of λ, α(λ) is absorption coefficient, *c* is the vacuum speed of light, and *ħ* is Planck’s constant. The refractive index, *n*, was determined from the Sellmeier dispersion equation [38].

Deriving the J–O parameters involved solving a set of four equations for transitions between J and J′ manifolds using the experimental line strengths. This simultaneous calculation, is based on Expression (1) and the electric dipole line strength:

(2) $$S_{JJ^{\prime }}^{ed}=\sum_{t=2,4,6}\Omega_{t}{\left|\left\langle \left(S,L\right)J|\left|U^{\left(t\right)}\right||\left(S^{\prime },L^{\prime }\right)J\prime \right\rangle \right|}^{2}$$

and the magnetic dipole (md) line strength:

(3) $$S_{JJ^{\prime }}^{md}={\left|\left\langle \left(S,L\right)J|\left|L+2S\right||\left(S^{\prime },L^{\prime }\right)J\prime \right\rangle \right|}^{2}$$

where $\left\langle \;|\left|U^{\left(t\right)}\right||\;\right\rangle$ are the reduced matrix elements of rank *t* (*t* = 2, 4, and 6) of tensor operators between states characterized by the quantum numbers (*S*, *L* and *J*) and (*S′*, *L′* and *J′*). We have used the values of the reduced matrix elements for the chosen Er^3+^ transitions from those tabulated in the work of Kaminskii [39]. Only the ^4^I_15/2_→^4^I_13/2_ transition has a magnetic dipole contribution corresponding to the absorption band around 1530 nm. The calculated line strength in this case is the sum of electric and magnetic dipole line strength. The calculated J-O parameters, by applying a least-squares fitting of *S*_meas_ and *S*_calc_, and the spectroscopic quality factor, χ, are given in Table 2. A comparison of the obtained J–O parameters with those obtained for BaF_2_:ErF_3_ [1,12,13,20,21], for BaF_2_:(Er,Yb) reported by [25] and for Er:YAG [40], is also provided. The observed variations between the Judd–Ofelt parameters obtained in this work and those reported in previous studies on BaF_2_:Er^3^⁺ or BaF_2_:(Er^3^⁺,Yb^3^⁺) systems can be attributed to several factors. Firstly, differences in dopant concentrations affect the local field environment and may lead to the formation of clusters rather than isolated centers, thereby altering the local symmetry and affecting the oscillator strengths [13]. Secondly, in co-doped systems such as the present one, additional energy transfer pathways and local charge compensation mechanisms are introduced due to the presence of both Er^3^⁺ and Yb^3^⁺ ions. This can modify the effective transition probabilities compared to a singly doped host. Thirdly, spectral overlap, especially in the near-UV region, may introduce uncertainties in the evaluation of certain bands; for instance, the ^4^I_15/2_ → ^4^G_11/2_ transition of Er^3^⁺ at ~378 nm overlaps with the 4f^14^→4f^13^5d transition of Yb^2^⁺, complicating the spectral deconvolution. Such overlapping transitions were excluded from the fit to ensure reliability of the extracted parameters.

The root mean square deviation, defined by $\Delta S_{rms}={\left[{\left(q-p\right)}^{-1}\sum {\left(S_{\mathrm{calc}}-S_{\mathrm{meas}}\right)}^{2}\right]}^{1/2}$ is a measure of the accuracy of the fitting procedure; *q* = 4 is the number of analyzed spectral bands and *p* = 3 is the number of sought parameters. In this case, Δ*S*_rms_ = 0.08·10^−20^ cm^2^, which is comparable with those obtained in other papers [1,12,13,28,40]. In order to calculate the radiative lifetime (τ_rad_) for an excited state, J′ can be used the relationship τ_rad_ = 1/∑*A*_JJ′_, where:

(4) $$A_{JJ^{\prime }}=\frac{64\pi^{2}e^{2}}{3\hbar (2J+1)\lambda_{mean}^{3}}\left[\frac{n{\left(n^{2}+2\right)}^{2}}{9}S_{JJ^{\prime }}^{ed}+n^{2}S_{JJ^{\prime }}^{md}\right]$$

is the sponaneous emission probability and the sum is taken over all final lower-lying states, $S_{JJ^{\prime }}^{ed}$ and $S_{JJ^{\prime }}^{md}$ are defined by Equations (2) and (3). The calculation of fluorescence branching ratios, B_JJ^′^_ involves utilizing the expression B_JJ^′^_ = *A_JJ^′^·_τ*_rad_. Table 3 shows the values of the radiative emission probabilities, the branching ratios, and the radiative lifetimes for transitions where luminescence was observed. A comparison of the calculated radiative lifetimes and those measured by other authors is also given in Table 3. The observed difference between calculated and measured lifetimes may imply the presence of energy migration, intense emission reabsorption or thermal coupling across manifolds, factors not taken into account in the standard J-O model.

### 3.3. Emission Spectra

**Figure 3:**
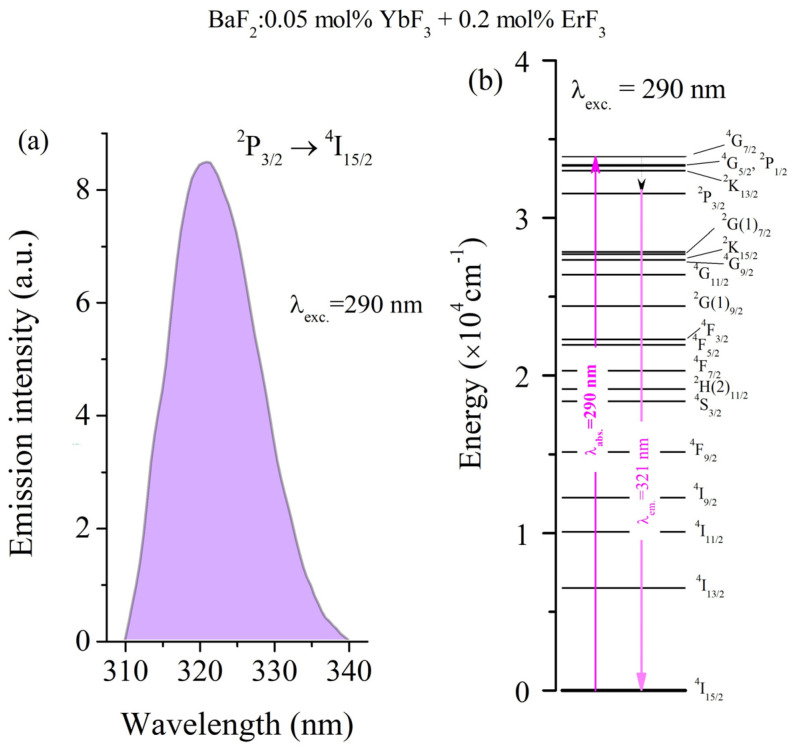
(**a**) Room temperature emission spectrum of BaF_2_: (0.05 mol% YbF_3_, 0.2 mol% ErF_3_) crystal under excitation at λ_exc_ = 290 nm. (**b**) The emission mechanism of Er^3+^ ions at λ_exc_ = 290 nm.

In order to obtain the room temperature emission spectra, two absorption bands were used for excitation, namely λ_exc._ = 290 nm (^4^I_15/2_→^4^G_7/2_ transition) and λ_exc._ = 378 nm (^4^I_15/2_→^4^G_11/2_ transition). The emission spectrum of the studied sample under 290 nm excitation and the energy level diagram are shown in Figure 3a,b. By excitation at 290 nm, the emission spectrum is characterized by an emission band centered around 321 nm (Figure 3a). This UV emission was reported in our previous work for various concentrations of simple-doped ErF_3_ doped BaF_2_ crystals [13] but it was not reported before in double-doped BaF_2_:(Er,Yb).

This emission can be attributed to the self-trapped exciton (STE). When the energy level ^4^G_7/2_ is pumped, the Er-bond exciton emission took place from the ^2^P_3/2_ manifold, which is placed in the middle of the STE emission band, therefore the energy transfer from STE to the Er^3+^ ion becomes effective [13].

**Figure 4:**
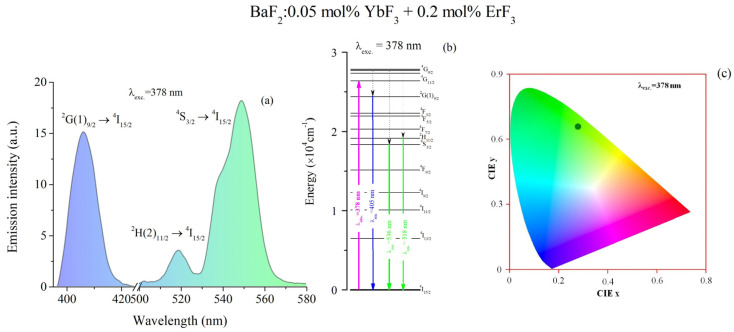
(**a**) Room temperature emission spectrum of BaF_2_: (0.05 mol% YbF_3_, 0.2 mol% ErF_3_) crystal under excitation at λ_exc_ = 378 nm. (**b**) The emission mechanism of Er^3+^ ions at λ_exc_ = 378 nm. (**c**) CIE chart for the emissions obtained under 378 nm excitation.

By excitation at 378 nm, we obtained three broad emission bands (Figure 4a). The green band has two peaks at 536 nm and 518 nm (weak) which correspond to the transition ^4^S_3/2_→^4^I_15/2_ and ^2^H(2)_11/2_→^4^I_15/2_ of Er^3+^ ions (Figure 4b). The blue band, around 405 nm, corresponds to the ^2^G(1)_9/2_→^4^I_15/2_ transition. The intensities of the blue and green emissions are over two times higher than the UV emission band. The International Commission on Illumination (CIE) chart corresponding to the visible emissions is shown in Figure 4c. The CIE coordinates were obtained using Gocie V2 software [41]. For the excitation at 378 nm, the color coordinates are X = 0.28 and Y = 0.66. These values are in a good agreement with those obtained for BaF_2_:0.15 mol% ErF_3_ (X = 0.30 and Y = 0.67) [13].

### 3.4. Dielectric Relaxation

**Figure 5:**
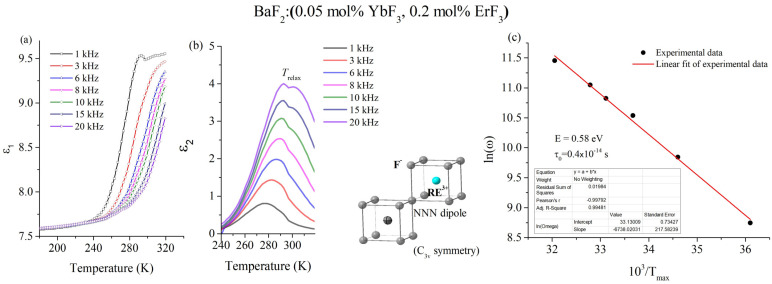
(**a**) Dielectric spectra of real part, ε_1_, and (**b**) imaginary part, ε_2_, of the complex dielectric constant at various frequencies ranging 1 ÷ 20 kHz (inset: *C*_3v_ site-symmetry), (**c**) determination of the relaxation parameters, *E* and τ_0_.

Figure 5a,b show the temperature and frequency variations of both the real, ε_1_, and imaginary, ε_2_, components of the complex dielectric constant, ε*, specifically for the BaF_2_:(0.05 mol% YbF_3_, 0.2 mol% ErF_3_) crystal. In the studied temperature range, the imaginary part (ε_2_) of the complex dielectric constant exhibits a singular peak at a temperature denoted as T_relax_, consistent across all frequencies. The T_relax_ temperature demonstrates a tendency to shift towards higher temperatures with increasing frequency, which indicates a dielectric relaxation phenomenon. The fundamentals of dielectric relaxation and the interpretation of relaxation phenomena in complex systems are well detailed in recent works [42,43,44]. The relaxation process is characterized by the activation energy for re-orientation, E, and by a relaxation time, τ, given by the Arrhenius relation τ = τ_0_ exp (E/kT), where τ_0_ is [45] the reciprocal frequency factor. The imaginary part, ε_2_, has a maximum for ωτ = 1 [45]. Therefore, the plot of 1/T_relax_ versus ln ω permits to determine the activation energy, E, and the τ_0_ (Figure 5c). The values of the relaxation parameters for the observed dipoles are E = 0.58 eV and τ_0_ = 4.1 × 10^−15^ s. These values correspond to the NNN dipoles (trigonal, C_3v_) sites (inset of Figure 5b). The RE^3+^ ions (erbium or ytterbium) substitute for Ba^2+^ ions in BaF_2_ crystals. Charge compensation is achieved by a fluorine ion in trigonal site symmetry. This result is in good agreement with our recent study regarding the analysis of site symmetries of Er^3+^ doped CaF_2_ and BaF_2_ crystals by high resolution photoluminescence spectroscopy [13]. For comparison, the relaxation parameters of Yb/Er/Gd doped BaF_2_ crystals are given in Table 4. Although the activation energy of 0.58 eV is consistent with values attributed in previous works to NNN dipoles of trigonal (C_3v_) symmetry in rare-earth doped BaF_2_ crystals, the potential contribution of other defect configurations cannot be entirely ruled out. Defect clusters or residual oxygen-related complexes may lead to similar relaxation signatures, especially at higher doping levels or under different growth atmospheres. Nevertheless, the low dopant concentrations employed here, and the close match to known C_3v_-related values, support our current assignment [13,27]. Further studies using EPR or impurity profiling would help to clarify the role of such alternative configurations.

The number of dipoles, *N*_D_, that contribute to the dielectric relaxation peak can be calculated from the dielectric spectra using the Campos method [46] that consists of plotting (*T* tan δ) versus 1000/*T*, where *T* is the temperature and tan δ is the dielectric loss. The maximum of this curve occurs when ωτ = 1, which corresponds to

(5) $$N_{D}=\frac{6\varepsilon_{0}k}{\mu^{2}}{\left(Ttan\delta \right)}_{max}$$

where *μ* = 8.596 × 10^−29^ C·m is the dipole moment of the NNN dipole, *k* is the Boltzmann constant, ε_0_ is the free space dielectric constant, and *N*_D_ is the number of dipoles contributing to the relaxation.

**Table 4:** Relaxation parameters of NNN dipoles in Yb/Er/Gd doped BaF_2_ crystals.

Sample	*E* (eV)	τ_0_ (10^−15^ s)	*N_D_* (10^17^ cm^−3^)
BaF_2_:0.05 mol% YbF_3_, 0.2 mol% ErF_3_	0.58	4.1	25.2 This work
BaF_2_:0.2 mol% ErF_3_	0.56	6.7	22.6 [26]
BaF_2_:0.05 mol% YbF_3_	0.53	16	20.95 [27]
BaF_2_:0.05 mol% GdF_3_	0.53–0.61	7 [47]	-

**Figure 6:**
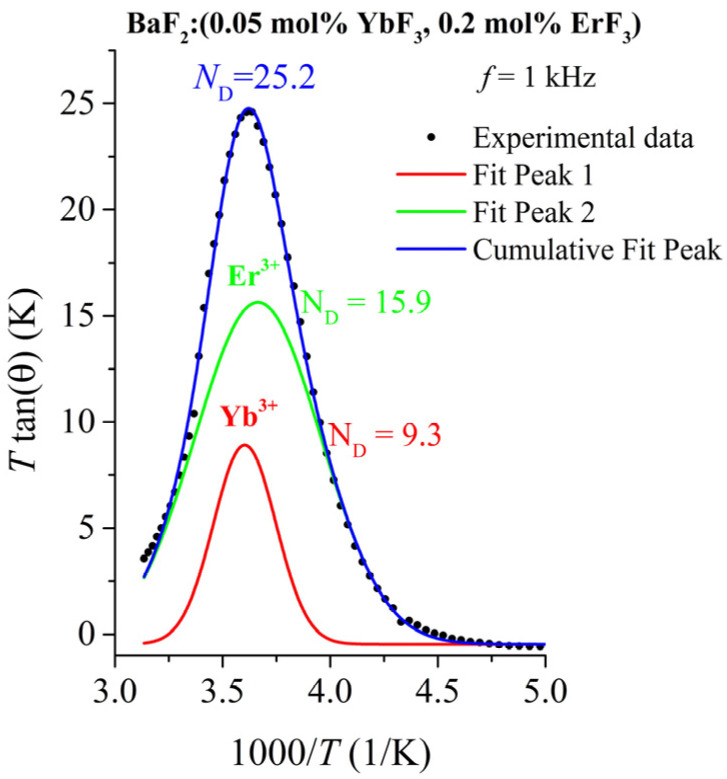
Plot of (*T* tan δ) versus 1000/T for *f* = 1 kHz.

After the Gaussian multi-peak fit of the plot (*T* tan δ) versus 1000/*T* for a frequency *f* = 1 kHz, the values of the *N_D_* for both RE ions can be estimated (Figure 6). In Table 4, the values for the concentration of NNN dipoles are shown. The NNN dipole concentration of Er^3+^ ions is 15.9·10^17^ cm^−3^ being slightly lower than for simply doped BaF_2_:Er crystals (see Table 4) [26]. Similarly, a lower NNN dipole concentration value of 9.3·10^17^ cm^−3^ was obtained for Yb^3+^ ions [27]. This can be explained by the clustering process in the double-doped crystal. No detailed study on the dielectric spectra of double-doped BaF_2_:(ErF_3_, YbF_3_) crystals has been previously reported.

## 4. Conclusions

A double-doped BaF_2_:(ErF_3_, YbF_3_) crystal was grown by using the conventional Bridgman technique. The optical and dielectric properties of the crystal were investigated. The significant results found in the present work are as follows: (1) The optical absorption spectrum shows both the characteristic bands of trivalent Er and Yb ions and the characteristic bands of Yb^2+^ ions (in the UV spectral range). (2) Using the J-O formalism, the radiative emission probabilities, the branching ratios, and the radiative liftimes were calculated. (3) Room temperature emission spectrum of BaF_2_:(0.05 mol% YbF_3_, 0.2 mol% ErF_3_) crystal under excitation at 290 and 378 nm was measured. (4) In the investigated temperature range, only one type of dielectric relaxation has been observed. This relaxation, with activation energy of 0.58 eV is associated with trigonal NNN centers. (5) The number of dipoles corresponding to NNN centers was determined. The values obtained are in agreement with those observed in singly doped crystals. The study of the dielectric behavior and optical properties of double-doped BaF_2_ crystal with low concentrations of YbF_3_ and ErF_3_ has not been reported so far.

In summary, the co-doped BaF_2_:Er^3^⁺,Yb^3^⁺ single crystal investigated in this work demonstrates a combination of favorable optical and dielectric properties, including well-resolved emission bands, radiative lifetimes in the sub-millisecond range, and a clearly defined dielectric relaxation process The Judd–Ofelt analysis reveals a relatively high Ω_2_ parameter and quality factor (χ = 1.41), indicating good radiative efficiency. These results highlight the suitability of such crystals for UV–visible photonic applications, particularly in compact solid-state luminescent sources. The findings contribute to the growing interest in rare-earth doped fluorides as multifunctional materials combining optical and dielectric functionalities.

## Figures and Tables

**Table 1:** The mean wavelength, the wavelength range, and the integrated absorption cross-section of the measured and calculated absorption line strengths of the selected absorption peaks of BaF_2_: (0.05 mol% YbF_3_, 0.2 mol% ErF_3_) crystal.

Trasition, ^4^I_15/2_ *→*	λ_mean_ (nm)	Wavelength Range (nm)	∑=∫α**(*λ*)**dλ (×10^−20^ cm^2^·nm)	$S_{DE}^{meas}$ (×10^−20^ cm^2^)	$S_{DE}^{calc}$ (×10^−20^ cm^2^)
^4^I_13/2_	1530	1450–1600	14.353	0.405	0.444
^4^F_9/2_	648	620–695	1.576	0.309	0.330
^2^H(2)_11/2_	518	502–533	2.471	0.604	0.603
^4^F_7/2_	486	476–498	1.271	0.331	0.226

**Table 2:** The Judd–Ofelt parameters, Ω_t_ (expressed in 10^−20^ cm^2^) and the spectroscopic quality factors, χ.

Ω_t_ (10^−20^ cm^2^)	BaF_2_: 0.2 mol% ErF_3_ + 0.05 mol% YbF_3_ (This Paper)	BaF_2_: 0.15 mol% ErF_3_ [13]	BaF_2_: 0.2 mol% ErF_3_ [12]	BaF_2_:Er^3+^ [1]	BaF_2_: 1.96 mol% ErF_3_, 2.91 mol% YbF_3_ [25]	YSAG: Er^3+^ [40]
Ω_2_	0.58983 ± 0.08026	0.6749	0.932	1.98	0.95	0.4681
Ω_4_	0.38250 ± 0.05751	0.1118	0.153	1.18	0.49	0.8378
Ω_6_	0.27115 ± 0.12981	0.5525	1.074	1.20	1.45	0.6741
χ = Ω_4_/Ω_6_	1.41	0.20	0.14	0.98	0.34	1.24

**Table 3:** The radiative emission probabilities, the branching ratios, and the calculated and measured radiative lifetimes for transitions where luminescence was observed.

Transition	λ_em_. [nm]	*A_JJ′_* (s^−1^)	β	*τ*_rad_ (ms) This Work	*τ*_exp_ (ms)
^4^S_3/2_→^4^I_15/2_	536	191.8	0.66	3.4	1.9 [13]
					0.96 [12]
					0.38 [19]
					0.56 [28]
^2^H(2)_11/2_→^4^I_15/2_	518	733.4	0.88	1.9	1.351 [13]
					0.96 [12]
					0.39 [19]
^2^G(1)_9/2_→^4^I_15/2_	405	209.1	0.42	2.0	-
^2^P_3/2_→^4^I_15/2_	321	79.2	0.09	1.1	0.906 [13]

## Data Availability

The data presented in this study are available on request from the corresponding author. The data are not publicly available due to privacy reasons.
